# Frequent attenders at primary care out-of-hours services: a registry-based observational study in Norway

**DOI:** 10.1186/s12913-018-3310-8

**Published:** 2018-06-25

**Authors:** Hogne Sandvik, Steinar Hunskaar

**Affiliations:** 1 National Centre for Emergency Primary Health Care, Uni Research Health, Kalfarveien 31, 5018 Bergen, Norway; 2 Department of Global Public Health and Primary Care, University of Bergen, Kalfarveien 31, 5018 Bergen, Norway

**Keywords:** Norway, Out-of-hours medical care, Emergency care, Primary health care

## Abstract

**Background:**

Out-of-hours (OOH) services are often consulted for problems that are non-urgent. Some of these patients are frequent attenders (FAs) who may constitute a heavy burden on the OOH service. The aim of the present study was to analyse FAs in a comprehensive material, covering all patients who have visited OOH services in Norway during a 10-year period.

**Methods:**

FA was defined as a patient having ≥5 consultations during one year. A cohort of all 15,172 FAs in 2008 was followed until 2017, with a description of demographics, consultations, and diagnoses for each year. FAs in 2017 were also analysed with more extreme definitions (≥10, ≥20, ≥30 consultations). To analyse predictors for FA a logistic regression analysis was performed on the 2017 data.

**Results:**

FAs constituted 2% of all patients (U-shaped age curve and female overrepresentation) and approximately 10% of all consultations each year. 59.8% of the cohort was never FA again, 17.7% had one relapse, 8.6% two, and 4.4% had three relapses. 22.8% was also a FA in 2009. Thereafter the percentage gradually declined to 6.2% in 2017. Only 0.8% of the original cohort were persistent FAs throughout the 10-year period. FAs were three times as likely to be given a psychological diagnosis as the average OOH patient, and this percentage increased in persistent and more extreme FAs. FAs tended to seek help at inconvenient hours (late evening and night), and increasingly so the more extreme they were. Also, they needed more consultation time and more often received home visits. The logistic regression analysis identified the following predictors for becoming FA (odds ratio = OR): Female (OR 1.17), age 0–1 years (OR 3.46), age 70+ (OR 1.57), small municipality (OR 1.61), psychological diagnosis (OR 10.00), social diagnosis (OR 5.97), cancer (OR 6.76), diabetes (OR 4.65), and chronic obstructive pulmonary disease (OR 7.81).

**Conclusions:**

FAs were most common among the youngest children and among the elderly, increasing with age. Females were overrepresented, as were patients with psychosocial problems and various chronic somatic conditions. The majority were only temporary FAs.

## Background

It is not uncommon for patients to seek help out of hours (OOH) for problems that are non-urgent, putting unnecessary strain on the OOH service [1, 2]. A non-urgent consultation may be defined as a consultation that could have waited until office hours the next day [1, 2]. Special attention has been given to so called frequent attenders (FAs) [1, 3–6]. Patients with medically unnecessary contacts are more often FAs [1]. Many studies on FAs have been performed in general practice and hospital emergency departments [7–18]. There is a strong association between frequent attendance OOH and frequent attendance during daytime general practice [8].

Different definitions have been used to identify FAs, making it difficult to compare the findings. In a review paper of FAs in general practice (published in 2005) Vedsted & Christensen identified 54 studies where the proportion of FA (with heterogeneous definitions) ranged from 3 to 25% of patients, or 2–24 contacts during varying time intervals [7]. Some included telephone contacts, others did not. When the top decile of the attenders was used as a definition of FA, it encompassed 30–50% of all contacts.

Studies of FAs in OOH services have also used different definitions. In a Danish study in 1990 the top decile was used as definition of FA, encompassing 42% of all contacts [3]. Another Danish study in 1997–98 included patients with ≥5 contacts during one year [5]. In a Dutch study in 2007 two definitions were used, patients with 3–6 contacts during a year were labelled FA (9% of attenders), those with ≥7 contacts VFA (very frequent attender, 1% of attenders) [4]. In another Dutch study in 2009–12 FA was defined as patients with ≥3 contacts during one year [1]. In 2011 an Italian study defined FA as patients with ≥3 contacts (including telephone contacts) during one year [6].

A majority of FAs in general practice and OOH tends to be female and elderly [1, 7, 14], while there is a male dominance among FAs in hospital emergency departments [12, 15, 16, 18]. Only rarely have children been included in studies [6]. Usually, FAs are characterized by psychological disorders [4, 6, 10–13, 19–21] and social disorders [10, 22, 23], but also chronic somatic disease has been reported as more common among FAs [10, 11, 20].

In follow-up studies a typical finding is that most frequent attendance is temporary [3, 4, 9–11, 17, 18]. A medical problem may develop over time and require several contacts. This is normal and should not be labelled inappropriate or unnecessary. However, it may be considered inappropriate use of OOH services. Persistent FAs are often characterized by psychological, social and addictive disorders, and chronic somatic disease [10, 11, 17, 18, 24].

Most studies of FAs are limited to single or a few locations, selected age groups, and short time periods. Few of them are from OOH services. It is likely that many FAs would be better taken care of by their regular general practitioner, rather than a random OOH doctor. Even being few, the FAs exert a heavy work load on the OOH services, and many have problems that are not well suited for a one-encounter-only service like the OOH. Detailed knowledge about FAs may thus help us to develop better services, both for patients and providers, and identify factors that may be target for intervention.

The aim of the present study was therefore to analyse frequent attenders, very frequent or extreme attenders, and persistent frequent attenders in a comprehensive material, covering all patients who have visited OOH services in Norway during a 10-year period.

### Organization of Norwegian health care

In Norway the state is responsible for hospitals, while the primary health care system is the responsibility of more than 400 municipalities. Primary care physicians (regular general practitioners, RGPs) are organized in a list system, to which more than 99% of the inhabitants subscribe. On average, each RGP cares for approximately 1100 inhabitants. RGPs are gate keepers, patients cannot decide by themselves to go to a hospital or emergency department.

OOH services are also the responsibility of municipalities. Some municipalities have their own OOH service, others cooperate. In 2016 there were 182 different OOH services in Norway, 81 municipal and 101 intermunicipal cooperatives [25]. OOH services are mainly staffed by RGPs, but other physicians may also participate.

OOH services are based on fee for service. As for out of pocket expenses, children younger than 16 years pay nothing. Others pay a little more than 30 USD for a consultation at the clinic and 40 USD for a home visit. FAs will often pay nothing since there is a limit (approximately 275 USD) to how much an individual have to pay for health services during one calendar year. In addition, the doctor or the OOH service sends an electronic compensation claim to the Norwegian Health Economics Administration (HELFO). Thus, HELFO has complete records of all patient contacts with OOH services in Norway. Compensation claims are time stamped and include a diagnosis according to ICPC-2 (International Classification of Primary Care, 2nd edition) [26]. There are different fee codes for different types of contact and for numerous different procedures. A time fee is claimed when the consultation lasts more than 20 min.

## Methods

The National Centre for Emergency Primary Health Care publishes annual statistics based on OOH-data provided by HELFO [27]. These data are anonymized, but since 2008 HELFO has included a pseudo-id based on the patient’s ID-number. This enables us to follow individuals’ use of OOH services through consecutive years. Due to privacy, the name of the individual municipality is not included in the material, but the municipalities are categorized into five groups based on the number of inhabitants.

All Norwegian citizens are given a unique personal identification number (ID-number) at birth. This number is used in various official records, including HELFO. Foreigners moving to Norway to stay for more than six months are also given an ID-number. As a rule all medical services will register a patient’s ID-number. However, in emergency settings these numbers are not always available.

The present study was based on data used for the annual statistics, but we included only face-to-face consultations with a doctor at the OOH clinic or during a home visit. Pseudo-id was lacking in 22.2% of all consultations in 2008, gradually improving until 2017, when only 1.8% of the consultations were without pseudo-id. The diagnostic distribution among consultations with or without pseudo-id varied slightly (data not shown).

The number of consultations remained stable throughout the period. There were 1,402,452 consultations in 2008 and in 2017 there were 1,399,001. The highest number was recorded in 2012 (1,436,297 consultations). As we have only included consultations with a pseudo-id, our material includes more consultations in 2017 (1,373,987 consultations) than in 2008 (1,091,483 consultations). The population of Norway was approximately 4.75 million in 2008 and 5.25 million in 2017.

We defined a frequent attender (FA) as a patient with five or more OOH consultations during one calendar year. By this definition, FAs made up 2% of all patients all years 2008–2017, accounting for approximately 10% of all consultations.

We established a cohort of all FAs found in 2008 and followed them until 2017. We recorded how many of the cohort patients were still frequent attenders in each year, and the number of consultations all cohort patients had in each year. Patients’ gender and age group were also recorded, in addition to the diagnostic distribution according to ICPC-2 chapters. A similar, separate analysis of psychological diagnoses among cohort patients was also performed.

The material from 2017 was subjected to a more detailed analysis. We used different definitions of frequent attenders (≥5, ≥10, ≥20, ≥30 consultations) and recorded diagnostic distribution, number of patients, consultations, gender, age, percentage of home visits, percentage of long consultations (> 20 min), and distribution of contacts during day, night, and week.

A logistic regression analysis was performed with being a FA (≥5 consultations) in 2017 or not as the dependent variable. Explanatory variables were gender, age group, number of inhabitants in the municipality (five groups), and five different diagnostic groups:

1. Psychological (ICPC-2 chapter P)
2. Social (ICPC-2 chapter Z)
3. Diabetes (ICPC-2 codes T89, T90)
4. Chronic obstructive pulmonary disease (COPD) (ICPC-2 code R95)
5. Cancer (ICPC-2 codes A79, B72, B73, B74, D74, D75, D76, D77, F74, H75, K72, L71, N74, R84, R85, S77, T71, T72, T73, U75, U76, U77, W72, X75, X76, X77, Y77, Y78)

The two chronic somatic diagnoses (diabetes and COPD) were chosen because they are prevalent and may cause complications or exacerbations. Individual patients were labelled with these diagnoses if they had at least one OOH contact with the relevant ICPC-2 code in 2017.

Since the material encompasses all reimbursement claims and not a sample, the differences identified are real and not fraught with statistical uncertainty. The data are therefore presented without confidence intervals and no statistical tests have been undertaken, except for the logistic regression analysis.

## Results

### The 2008 cohort

In 2008 there were 1,091,483 consultations with 746,241 patients identified by pseudo-id (1.5 consultations per patient). Of these, 15,172 patients were identified as FAs and included in the cohort (Table 1). The majority (59.8%) of the cohort patients were never FA again during the following 10-year period.

**Table 1:** Characteristics of all identified OOH patients in 2008 and a cohort of frequent attenders established in 2008

		Cohort of frequent attenders in 2008 (*n* = 15,172 patients)									
	All 2008	2008	2009	2010	2011	2012	2013	2014	2015	2016	2017
Number of patients who have consulted at least once during the year	746,241	15,172	11,508	9690	8745	7859	7197	6614	6196	5772	5421
Percentage frequent attenders of cohort each year	2.0	100.0	22.8	16.3	13.3	11.0	9.6	8.5	7.8	6.7	6.2
Percentage frequent attenders of cohort every year since 2008	2.0	100.0	22.8	9.9	5.5	3.7	2.4	1.8	1.3	1.0	0.8
Consultations per patient	1.5	7.0	4.4	4.0	3.8	3.6	3.5	3.5	3.4	3.3	3.2
Percentage females	52.5	55.7	56.8	58.1	58.8	60.3	60.6	61.1	61.3	62.0	61.6
Percentage aged 0–1 in 2008	4.4	10.3	11.0	10.8	10.4	9.6	9.1	8.9	8.9	8.6	8.4
Percentage aged 2–15 in 2008	16.0	10.0	9.8	9.5	9.6	9.5	9.2	9.3	9.5	10.1	9.8
Percentage aged 16–69 in 2008	66.5	59.9	61.9	65.1	67.0	69.5	71.6	72.8	73.7	74.3	76.0
Percentage aged 70+ in 2008	13.1	19.9	17.3	14.6	13.0	11.5	10.1	9.0	7.9	7.0	5.8

In 2009 22.8% of the patients in the 2008 cohort were still FAs. During the next years the percentage of FAs in the 2008 cohort gradually declined to 6.2% in 2017. Likewise, the average number of consultations per patient in the cohort declined from 7.0 to 3.2, still more than twice the average number of consultations among all OOH patients. The percentage of cohort patients who remained persistent FAs *every* year since 2008, declined from 22.8% in 2009 to 0.8% in 2017 (Table 1).

Table 1 also demonstrates that females were overrepresented among FAs, and even more so among cohort patients who continued to visit the OOH services during the following years. The age distribution in the cohort changed over the years. Those who were ≥ 70 years old in 2008 gradually decreased, while the age group 15–69 years increased. Only a small decrease was observed among those who were 0–1 years in 2008.

There were 124 patients who remained persistent FAs every year during the 10-year period. Two thirds of them were women, mean age was 41.2 years in 2008, median 39.5, and range 16–84. In 2008 22.2% of their consultations were for psychological and 1.2% for social diagnoses. In 2017 the corresponding numbers were 24.5% psychological and 2.0% social diagnoses.

Of the 15,172 cohort patients 17.7% had one relapse as FA, 8.6% had two, and 4.4% had three relapses during the following 10-year period. Table 2 demonstrates that the cohort patients had an increased risk of being FA until 2012, after which the risk was less than 2%. The table also shows that there was an increased risk of being FA again the first years after a relapse, especially if the relapse took place during the first years.

**Table 2:** Overview of patients in the original 2008 cohort of 15,172 frequent attenders (FAs) who had their first relapse as FA the following years. For each year the table shows the percentage who were never FA again after their first relapse and the percentage who were FA the following years

				Percentage FA each year after first relapse as FA							
	N	Percentage of the original cohort (*n* = 15,172)	Never FA again (%)	2010	2011	2012	2013	2014	2015	2016	2017
First relapse as FA in 2009	3454	22.8	36.0	43.4	33.2	27.6	23.8	21.0	18.4	16.6	14.4
First relapse as FA in 2010	967	6.4	46.4		33.3	24.0	19.5	15.9	16.4	12.5	12.7
First relapse as FA in 2011	550	3.6	49.3			29.5	21.8	15.6	15.8	10.2	11.1
First relapse as FA in 2012	327	2.2	54.4				23.9	19.3	19.6	14.7	11.9
First relapse as FA in 2013	247	1.6	54.7					27.5	18.6	11.3	14.6
First relapse as FA in 2014	190	1.3	57.4						28.4	18.9	18.9
First relapse as FA in 2015	145	1.0	75.9							17.9	11.0
First relapse as FA in 2016	128	0.8	71.1								28.9
First relapse as FA in 2017	94	0.6	–								

In 2008 FAs were three times as likely to be given a psychological diagnosis as the average OOH patient. During the years after 2008 cohort patients were even more prone to receive a P-diagnosis. Social problems (Z-diagnoses) were also more prevalent among FAs (Table 3).

**Table 3:** Diagnostic distribution (percentage of consultations) in 2008 and in a cohort of frequent attenders in 2008 in subsequent years

		Cohort of frequent attenders in 2008 (*n* = 15,172 patients)									
*ICPC-2 chapter*	All 2008	2008	2009	2010	2011	2012	2013	2014	2015	2016	2017
A: General. unspecified	10.3	11.5	12.2	11.1	12.1	12.5	13.2	13.5	14.1	13.4	13.4
B: Blood etc.	0.3	0.4	0.3	0.3	0.3	0.3	0.3	0.2	0.3	0.3	0.3
D: Digestive	10.1	11.5	11.8	12.2	12.1	11.1	11.8	12.8	11.5	11.7	12.2
F: Eye	4.7	2.1	1.9	1.9	1.9	1.9	1.7	1.9	1.9	1.9	1.9
H: Ear	3.2	2.4	1.9	1.7	1.9	1.6	1.5	1.3	1.3	1.3	1.2
K: Cardiovascular	4.1	5.0	4.2	4.2	4.3	3.8	3.9	3.7	3.8	4.2	4.2
L: Musculoskeletal	16.3	11.3	11.8	12.8	13.4	13.3	13.6	13.2	14.3	14.9	14.2
N: Neurological	3.7	4.6	4.7	4.7	4.8	4.8	5.0	4.8	4.9	5.0	5.2
P: Psychological	4.3	12.9	15.6	16.9	16.1	17.3	18.3	17.5	17.4	16.8	16.4
R: Respiratory	22.2	20.9	20.0	18.1	16.9	17.3	15.1	14.4	14.0	14.7	14.8
S: Skin	11.2	6.3	5.8	6.3	6.2	5.9	6.0	6.4	7.0	6.7	6.9
T: Endocrine etc.	0.8	0.9	0.9	0.8	1.0	0.8	0.8	0.9	1.0	1.0	0.9
U: Urological	5.8	6.7	6.1	6.0	5.9	5.9	5.7	6.2	5.6	5.3	5.7
W: Pregnancy etc	1.3	1.2	0.9	0.9	0.9	0.8	0.9	0.7	0.9	0.6	0.6
X: Female genital	0.9	0.9	0.9	1.0	0.9	1.1	0.9	1.0	0.8	0.8	0.8
Y: Male genital	0.7	0.6	0.7	0.6	0.8	0.8	0.6	0.7	0.5	0.6	0.7
Z: Social problems	0.2	0.5	0.5	0.5	0.6	0.8	0.7	0.7	0.5	0.7	0.7
Number of consultations with identified patients	1,091,483	106,655	50,499	38,967	33,114	28,192	25,471	23,223	21,162	19,172	17,474

Table 4 shows the distribution of the most common psychological diagnoses among all patients and among FAs in 2008, and among the 2008 cohort patient in all years from 2008 to 2017. Chronic alcohol abuse, drug abuse, schizophrenia, and personality disorders were more common among FAs, and even more so among cohort patients in the following years.

**Table 4:** Distribution (percentage of consultations) of the most common psychological diagnoses in 2008 and in a cohort of frequent attenders in 2008 in subsequent years

		Identified patients with five or more OOH consultations in 2008 (frequent attenders in 2008)									
*ICPC-2 codes*	All 2008	2008	2009	2010	2011	2012	2013	2014	2015	2016	2017
P01: Feeling anxious, nervous, tense	5.3	5.5	5.4	4.4	4.0	5.7	4.4	6.9	8.5	9.8	11.0
P02: Acute stress reaction	7.1	4.3	3.1	3.8	3.6	5.0	4.2	2.7	3.1	2.9	3.3
P06: Sleep disturbance	3.2	2.5	2.2	1.9	2.2	2.1	2.0	1.5	1.5	1.8	1.9
P15: Chronic alcohol abuse	7.0	9.7	9.7	11.3	11.5	12.5	14.8	10.7	10.3	9.4	8.7
P16: Acute alcohol abuse	8.9	6.2	6.7	6.2	5.3	9.0	7.4	7.2	7.6	7.5	7.5
P19: Drug abuse	6.8	8.7	9.1	10.3	11.4	11.8	13.4	12.1	10.9	12.4	10.5
P72: Schizophrenia	3.9	5.5	5.6	6.5	6.7	6.6	7.1	6.2	4.6	5.0	5.1
P74: Anxiety disorder. Anxiety state	8.5	9.9	9.2	9.1	9.5	6.4	7.2	6.3	6.1	5.4	5.0
P76: Depressive disorder	12.9	10.9	10.0	9.3	8.7	5.6	5.3	6.0	5.7	5.1	5.4
P77: Suicide. Suicide attempt	3.5	3.6	3.6	4.0	3.7	4.0	4.2	5.4	6.0	7.4	6.8
P80: Personality disorder	1.4	3.1	3.3	3.8	3.8	2.6	3.6	5.1	5.3	4.9	5.7
P98: Psychosis, other	3.4	2.7	3.1	2.6	3.0	2.3	2.6	2.6	2.9	2.5	2.4
P99: Psychological disorder, other	5.4	7.7	9.9	7.9	6.7	8.6	6.7	8.6	7.2	5.3	8.2
Number of consultations with identified patients	47,189	13,771	7862	6570	5334	4868	4665	4059	3689	3213	2865

### The 2017 data

**Fig. 1:**
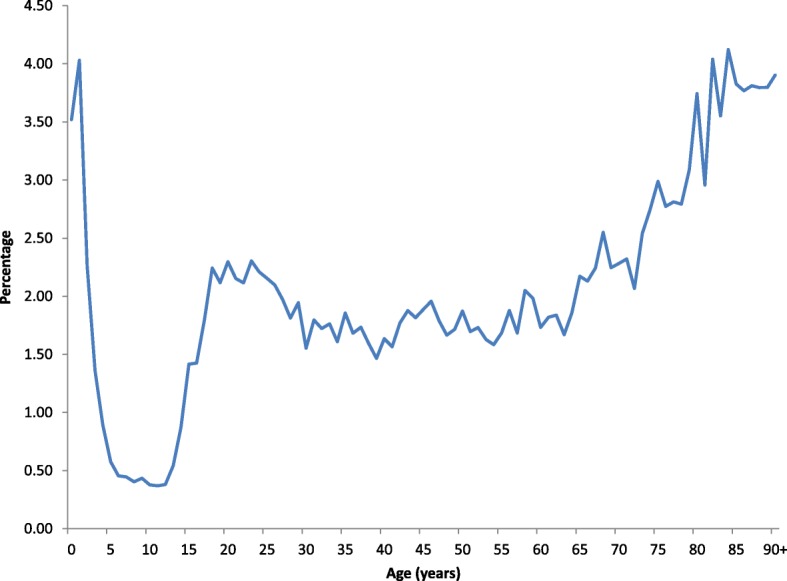
Percentage of frequent attenders (of all OOH patients) according to age in 2017

The percentage of FAs was high among the youngest children, but declined rapidly, reaching a low at age 10–12 years. Thereafter the percentage increased and stabilized around 2% until increasing again among the elderly (Fig. 1).

**Fig. 2:**
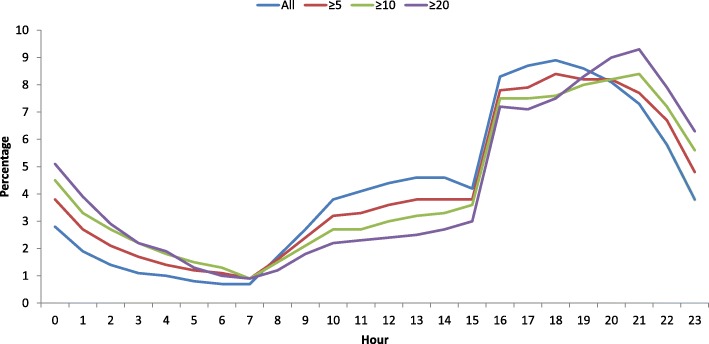
Distribution of OOH consultations during day and night for all patients, and for frequent attenders with ≥5 consultations, ≥10 consultations or ≥ 20 consultation in 2017

Increasing the threshold for defining FAs resulted in progressively more deviant patients’ characteristics. FAs were more prone to contact the OOH services during night and late evening, and more so when the FA definition was sharpened (Fig. 2). An opposite tendency was found for consulting during weekends (Table 5). With sharpened FA definition an increasing percentage of the patients were female, more home visits were performed, and the physicians claimed more time fees for long consultations. Table 5 also demonstrates that the percentage of psychological and social diagnoses increased considerably when the FA definition was sharpened.

**Table 5:** Diagnostic distribution (percentage of consultations) and characteristics of patients and consultations with different definitions of frequent attenders according to frequency of consultations (2017)

*ICPC-2 chapter*	All	≥5 consultations	≥10 consultations	≥20 consultations	≥30 consultations
A: General. unspecified	13.3	14.7	14.9	15.0	16.7
B: Blood etc.	0.3	0.4	0.5	0.7	0.9
D: Digestive	10.6	11.9	11.6	12.2	12.3
F: Eye	4.4	1.5	0.8	0.4	0.3
H: Ear	2.5	1.7	0.8	0.3	0.1
K: Cardiovascular	3.8	4.5	4.1	3.7	3.5
L: Musculoskeletal	16.6	9.7	8.2	6.2	4.7
N: Neurological	4.3	4.5	4.6	4.6	3.8
P: Psychological	5.0	16.3	28.0	33.6	34.0
R: Respiratory	18.5	17.5	11.4	7.4	7.5
S: Skin	11.1	6.3	6.2	8.2	9.5
T: Endocrine etc.	0.9	1.3	1.2	0.7	0.6
U: Urological	5.8	6.6	4.7	3.2	2.1
W: Pregnancy etc.	1.0	0.8	0.4	0.1	0.1
X: Female genital	0.8	0.7	0.6	0.5	0.1
Y: Male genital	0.7	0.6	0.6	0.9	1.2
Z: Social problems	0.3	0.9	1.4	2.3	2.3
Number of consultations with identified patients	1,373,987	136,773	38,820	13,398	6785
Number of patients	946,176	18,876	2462	429	150
Number of consultations per patient	1.5	7.2	15.8	31.2	45.2
Percentage females	51.6	54.8	56.3	63.4	63.3
Mean age	37.7	42.4	45.4	43.1	41.5
Percentage home visits	4.8	11.5	14.7	15.4	16.6
Percentage consultations during weekends	40.7	37.8	36.8	35.5	34.8
Percentage consultations with time fee	39.7	46.5	48.7	49.3	48.2

The logistic regression analysis showed that females were more likely to be FAs, as were children aged 0–1 years and the elderly. FAs were also more common in small municipalities. Both psychological, social and cancer diagnoses were strong predictors of becoming a FA, as were diabetes and COPD (Table 6).

**Table 6:** Odds ratio for being a frequent attender (*n* = 18,876) at OOH services in 2017 (*n* = 946,176 patients)

	N	Odds ratio	95% confidence interval
Gender			
Male (ref.)	457,965		
Female	488,211	1.17	1.13–1.20
Age groups			
16–69 (ref.)	590,010		
0–1	51,822	3.46	3.28–3.64
2–15	167,658	0.71	0.67–0.75
70+	136,686	1.57	1.51–1.64
Number of inhabitants in municipality			
> 50,000 (ref.)	337,545		
10,001–50,000	454,610	0.96	0.93–0.99
5001–10,000	83,370	1.23	1.17–1.30
2001–5000	57,123	1.27	1.19–1.34
≤ 2000	13,528	1.61	1.46–1.77
Psychological diagnosis (ref. category: all other)	46,988	10.00	9.66–10.36
Social diagnosis (ref. category: all other)	4025	5.97	5.45–6.53
Cancer diagnosis (ref. category: all other)	3811	6.76	6.09–7.51
Diabetes (ref. category: all other)	4383	4.65	4.18–5.18
Chronic obstructive pulmonary disease (ref. category: all other)	7828	7.81	7.27–8.39

## Discussion

We found that frequent attenders (FAs) were most prevalent among the youngest children and among the elderly, increasing with age. Females were overrepresented, as were patients with psychosocial problems and various chronic somatic conditions.

Most were only temporary FAs, almost 60% were never FA again during the 10-year period. Only 22.8% were also FA the next year (2009). After four years without relapse the risk for being a FA was at the same level as for the average OOH patient. This is in line with other studies [3, 4, 9–11, 17, 18]. Only 0.8% of the original cohort remained persistent FAs every year during the 10-year period. However, a higher percentage of the cohort patients were temporary FAs in some of the years, e.g. 6.2% in 2017.The average number of consultations per patient in the cohort remained high throughout the period, still more than twice the normal level in 2017. It may therefore be speculated that the FAs consist of two different groups of patients. One group has typically a temporary medical problem that requires several consultations during a relatively short period of time, while another group is prone to use OHH services for several reasons over a longer period of time, and thus develops habituation to OOH use for both urgent and non-urgent situations.

The age distribution in the cohort changed as expected over the years, with the exception of the youngest FAs (aged 0–1 years) who continued to consult frequently over the years, probably an effect of the parents’ help seeking behaviour [28]. It remains to see if these children continue with frequent attendance when they grow old enough to decide for themselves.

Over time the remaining 2008 cohort consisted of more female patients, and an increasing percentage of the consultations were for psychological and social problems. Other studies of persistent FAs have drawn similar conclusions. In a study from Dutch general practice persistence was predicted by psychological, social and addictive disorder, and chronic somatic disease, but not by gender and age [10, 11]. In an Australian community study persistence was associated with female gender, depression, disability and poor self-reported health [24]. A Swedish general practice study found that a large majority of persistent FAs were females [9]. A study of persistent FAs at a US university hospital emergency department found that persistent FAs had more psychiatric problems and substance abuse [17]. Similar findings have been reported from New Zealand [18].

Psychological problems are obviously a hallmark of frequent and persistent attendance [4, 6, 7, 12], but there are differences between single diagnoses. We found that some short term psychiatric problems are less common among FA, such as acute stress reaction, sleep disturbance or depressive disorder. On the other hand more chronic conditions such as schizophrenia, personality disorder, drug abuse and chronic alcohol abuse were clearly overrepresented among FAs and persistent FAs.

The characteristics of very frequent or extreme attenders (≥ 10, ≥ 20, and ≥ 30 consultations) were similar to persistent FAs, with a larger percentage of females and more psychological and social problems than ordinary FAs. This is in line with other studies of very frequent or extreme FAs [4, 12, 13]. We also found that extreme FAs tended to seek help at inconvenient hours (late evening and night), and increasingly so the more extreme they were. Also, they needed more consultation time and more often received home visits. It is no surprise that these patients can be a challenge for the doctor on call.

The logistic regression analysis confirmed the findings in the descriptive analyses. In addition, we found that FAs were more common in small municipalities. It is known that overall OOH contact rates are higher in rural municipalities than in urban areas [29]. OOH services in smaller municipalities care for fewer patients and the doctor on call is less busy than in more populous municipalities. Furthermore, it is often the same doctor who manages the patient both during working hours and OOH. OOH doctors in the smallest municipalities do home visits 4–6 times as frequently as doctors in larger municipalities (percentage of all contacts) [27]. It is quite possible that some of the OOH consultations in these municipalities are performed because they are simply more convenient for both patient and doctor than during working hours.

Chronic somatic illness have been found to predict frequent attendance in general practice [10, 11, 20], as well as in OOH services [6, 10]. We found that COPD and diabetes were more common among FAs than among ordinary attenders. Probably, complications and exacerbations of chronic conditions are the reasons for this help seeking behaviour.

A study from 2014 found that cancer patients received more home visits from the OOH services than non-cancer patients [30]. The same study concluded that there was no indication of overuse of OOH services by cancer patients in Norway. The present study leaves this conclusion in doubt. We found that there was almost a sevenfold chance for a cancer patient to become a FA compared with non-cancer patients. This finding indicates that there is a potential for improving the daily health care for cancer patients. It should be organized in such a way that there is less need for them to consult random OOH doctors.

More accessible health care services during office hours would probably reduce the pressure on OOH services. Half of all patients interviewed at an OOH service were willing to wait until the next day to see their personal physician, provided they were guaranteed an appointment [2]. It is quite obvious that not only cancer patients, but also patients with psychosocial problems are better served by a doctor who can provide a continuous relationship.

Future research should aim towards a better understanding of the help-seeking behaviour of FAs, especially those who consult repeatedly for non-urgent problems. Probably, qualitative methods would be a proper approach for this type of research. Interventions could be designed to reduce the need for consulting OOH services [5]. And for policy makers an important task would be to increase the capacity and accessibility of daytime general practice.

### Strengths and limitations

There is no well-defined definition of FA. We chose to use ≥5 consultations per year, while others have used both more or less. With our definition, 10% of all consultations were ascribed to FAs. We think this is a reasonable choice, and would argue against setting the threshold so low that almost half of all consultations are included. Furthermore, the size of the present material makes it possible to analyse even more extreme FAs with reasonable power.

Reimbursement claims are prepared for all contacts, and we may therefore assume that all OOH consultations have been registered. There is also reason to assume that the information on gender, age, municipality, time of inquiry and fee codes is correct.

Although formal criteria for the use of ICPC-2 diagnostic codes are available, it is not common to check these in daily clinical activities. This lack of diagnostic precision is likely to be evenly distributed through the material, and we believe that the differences between various groups and over time still remain valid, especially at ICPC-2 chapter level. The annual statistics also demonstrate stable diagnostic distribution for consultations from year to year [27].

In 2008 22.2% of the consultations had to be excluded because of lack of a pseudo-id. The diagnostic distribution among these differed slightly from those who were identified by pseudo-id. However, in 2017 only 1.7% of all consultations were unidentified.

Our data are probably representative for countries with similar health care systems, i.e. mainly tax financed, based on general practitioners, preferably organized in a list system, and with gate keeping for secondary care.

## Conclusion

Frequent attenders were most common among the youngest children and among the elderly, increasing with age. Females were overrepresented, as were patients with psychosocial problems and various chronic somatic conditions. The majority were only temporary frequent attenders. Persistent, very frequent or extreme attenders were characterized by an even larger percentage of females and more psychological and social problems. Probably, increased accessibility at daytime general practice would reduce the need for frequent use of OOH services. For a group of frequent attenders with mainly non-urgent encounters, there may be a need of an individually focused intervention in collaboration with the patient’s general practitioner and other primary care services in the municipality.

## Abbreviations

COPD: Chronic obstructive pulmonary disease
FA: Frequent attender
HELFO: Norwegian Health Economics Administration
ICPC-2: International Classification of Primary Care 2nd edition
OOH: Out of hours
RGP: Regular general practitioner
VFA: Very frequent attender
